# Incident reports involving hospital administrative staff: analysis of data from the Japan Council for Quality Health care nationwide database

**DOI:** 10.1186/s12913-020-05903-1

**Published:** 2020-11-20

**Authors:** Naomi Akiyama, Tomoya Akiyama, Kenshi Hayashida, Takeru Shiroiwa, Keisuke Koeda

**Affiliations:** 1 Iwate Medical University, 2-1-1 Idaidori, Yahabacho, Shiwagun, Iwate 028-3695 Japan; 2 Department of Medical Informatics and Management, University Hospital, University of Occupational and Environmental Health, Kitakyushu, Japan; 3 National Institute of Public Health (NIPH) Center for Outcomes Research and Economic Evaluation for Health (C2H), Wako, Japan

**Keywords:** Hospital administrative staff, Incidents, Medication error, Task shifting, Task sharing, Patient safety, Quality improvement, Risk management

## Abstract

**Background:**

Task shifting and task sharing in health care are rapidly becoming more common as the shortage of physicians increases. However, research has not yet examined the changing roles of hospital administrative staff. This study clarified: (1) the adverse incidents caused by hospital administrative staff, and the direct and indirect impact of these incidents on patient care; and (2) the incidents that directly involved hospital administrative staff.

**Methods:**

This study used case report data from the Japan Council for Quality Health care collected from April 1, 2010 to March 31, 2019, including a total of 30,823 reports. In April 2020, only the 88 self-reported incidents by hospital administrative staff were downloaded, excluding incidents reported by those in medical and co-medical occupations. Data from three reports implicating pharmacists were rejected and the quantitative and textual data from the remaining 85 case reports were analyzed in terms of whether they impacted patient care directly or indirectly.

**Results:**

Thirty-nine reports (45.9%) involved direct impact on patient care, while 46 (54.1%) involved indirect impact on patient care. Most incidents that directly impacted patient care involved administrative staff writing prescriptions on behalf of a doctor (*n* = 24, 61.5%); followed by errors related to system administration, information, and documentation (*n* = 7, 17.9%). Most reported errors that indirectly affected patient care were related to system administration, information, and documentation used by administrative staff (*n* = 22, 47.8%), or to reception (*n* = 9, 19.6%). Almost all errors occurred during weekdays. Most frequent incidents involved outpatients (*n* = 23, 27.1%), or occurred next to examination/operation rooms (*n* = 12, 14.1%). Further, a total of 14 cases (16.5%) involved patient misidentification.

**Conclusions:**

Incidents involving hospital administrative staff, the most common of which are medication errors from incorrect prescriptions, can lead to severe consequences for patients. Given that administrative staff now form a part of medical treatment teams, improvements in patient care may require further submission and review of incident reports involving administrative staff.

## Background

The rate of population aging and the number of older adults, defined as people aged 65 years and above, are increasing in some countries [1]. Japan faces serious policy problems regarding its aging society, including a shortage of physicians. In an effort to address these problems, the number of physicians per capita continues to rise with the goal of reaching the level recommended by the Organisation for Economic Co-operation and Development. The numbers of physician assistants and advanced practice registered nurses also continue to increase. However, the estimated supply of physicians will not fulfill the demand for health care in an aging society; to meet the projected needs, the number of new physicians must increase by 53% over the current pace [2]. Thus, the Japanese government has strategized a new solution to the problem of physician shortage as part of a series of actions for promoting work-style reform [3]. Comprehensively, the reforms aim to create greater stability in the national workforce by formalizing improvements to various aspects of employment in Japan, such as improved work-life balance and ensuring equal access to health care. To meet these goals, the reforms will allow for “a wide variety of work patterns” and “expand the duties of industrial physicians and strengthen the function of occupational health services.” [3]

Task shifting from physicians to nurses has created interest among policymakers as a strategy for responding to staffing shortages and increasing access to primary care; task shifting in this sense has been reported as efficient and cost-effective [4, 5]. This strategy of work reform in Japan is part of a global trend [3]. However, the long working hours of physicians continue to be a serious problem in Japan, prompting the government to provide incentives to hospitals that have adopted systems that shift some tasks to nurses and pharmacists. For example, the Ministry of Health, Labor and Welfare offered incentives for physicians’ offices adding work support systems. In the Ministry’s 2008 medical fee revision, a new qualification of medical assistant was introduced. For registration as a medical assistant, 32 accredited education hours related to medical care, followed by a qualifying examination are necessary [6]. Medical assistants are authorized to enter prescription information and predetermined examination orders onto patients’ charts on behalf of doctors. They are hired as hospital administrative staff and usually work in outpatient departments or hospital wards.

After registered nurses, who, at 38.5% of the total staff, comprise the largest number of employees per hospital, administrative staff and physicians are the second- and third-largest groups, accounting for 10.5 and 10.3% of all hospital staff, respectively, as of 2017 [7]. Yet previous studies have indicated that administrative staff in Japan report fewer incidents than nurses. However, few nationwide studies have described the roles of administrative staff in Japan, and many of them were case studies involving one hospital, such as Ishibashi et al. [8] and Osawa [9].

Previous studies of incident reports focused on reports involving clinical staff such as physicians, nurses, and pharmacists whose major roles directly impact patient care. However, the roles of hospital administrative staff encompass an indirect impact on patient care, which may be the reason why recent studies of incident reports have not focused on reports involving administrative staff, choosing instead to focus on roles with a direct impact on patient care. Therefore, the objective of this study was to examine incident reports involving hospital administrative staff to clarify: (1) the types of incidents reported, and (2) how situations involving incidents were reported, focusing on their direct and indirect impacts on patient care.

## Methods

### Operational definitions

Administrative staff in health care include clerks, medical secretaries, medical assistants, receptionists, and accountants. Direct care is care that directly impacts a patient, through the provision of services to a patient that require some degree of interaction between the patient and the health care provider. Examples include assessments, performing procedures, advising, and implementation of a care plan [10]. Indirect care is care that does not directly impact a patient; services that are related to patient care but do not require interaction between the health care provider and the patient. Examples include charting and scheduling [10]. It is common to divide care into direct and indirect care in the nursing field [11, 12].

### Study design

This study used secondary data from the Japan Council for Quality Health care (JCQHC). JCQHC collects all reports of adverse events and incidents (including errors and near-misses) nationwide from hospitals in Japan. The total number of reports was 1018 in 2015, with 275 mandatory reports and 743 voluntary reports from institutions. These institutions voluntarily collected the reports from their staff, and selected a portion of the reports to send to the JCQHC. In FY2004, the Division of Adverse Event Prevention began implementing the Project to Collect Medical Near-miss/Adverse Event Information, which gathers information about medical adverse events and medical near-miss events, with the objective of promoting safety in medical care. The JCQHC compiles the information gathered concerning medical adverse events and anonymizes it before releasing it to the public [13]. The JCQHC website contains open data, which have been used to study medical incidents in previous studies. For example, Akiyama et al. [14] used JCQHC data to compare differences in nurse-related incidents in terms of various factors, such as clinical experience level, drug administration, and medical devices used. Similarly, Ichikawa et al. [15] used JCQHC data to implement case analyses of reports of pediatrician-related medical adverse events. The data include the type of near miss, information about the person involved (e.g., job, years of clinical experience, and years working within a particular department), and contributing factors to the incidents.

### Data collection

Open data from the JCQHC were obtained for the nine-year period from April 1, 2010, to March 31, 2019, including a total of 30,823 reports [13]. These reports, which were voluntarily submitted by hospital staff, were sent from cooperating hospitals via the electronic system of the JCQHC every month. Data from the JCQHC were downloaded in April 2020, and included all incidents reported by hospital administrative staff, and excluded medical and major co-medical occupations such as physicians, nurses, dentists, rehabilitation therapists, and clinical technologists. All 88 self-reported incidents of hospital administrative staff were checked and it was ensured that the reports did not implicate other medical staff, such as nurses or physicians. Data from three of the reports pertaining to pharmacists were excluded, leaving a total of 85 incidents that were reported by hospital administrative staff, including medical assistants, medical clerks, and medical secretaries.

### Analysis

JCQHC data are both quantitative and textual. The quantitative data items include information regarding when (time and day) and where (area of the hospital) the incidents occurred, and who was involved (relevant personnel and their positions). Textual data items describe the reported incidents, such as the situation, what happened, the background, related factors, and the resulting improvement plan. The data in this study were categorized using content analysis, depending on whether the incident affected patient care directly or indirectly. Similarly, the type of incident was categorized as medication-related, surgery-related, exam and/or treatment-related, or other, as well as whether the incident involved patient misidentification. Work indirectly related to patient care, including the management of patient charts, is within the job description of administrative staff. Patient misidentification is defined as any instance of staff mistaking the identity of a patient or patient ID, or patients having ID cards that belong to other patients. Both quantitative and textual data were used to characterize the differences between direct and indirect effects on patient care using descriptive statistics. Textual data were analyzed using content analysis, which is well suited to analyzing multifaceted characteristics of phenomena in a sensitive manner. We focused on the case factors regarding the “what” and “why” of the reported incidents.

### Ethical considerations

This study was conducted using JCQHC data. These data are available as open access; sensitive information has been deleted by JCQHC staff and all information has been anonymized to prevent possible identification of individuals and facilities.

## Results

### Incident reports with direct impact on patient care

Thirty-nine out of 85 cases (45.9%) involved direct care to patients. These are presented by themes in Table 1. For direct-care incidents, four categories of themes were extracted: drug prescriptions (*n* = 24, 61.5%); system administration, information, and documentation (*n* = 7, 17.9%), inquiry (*n* = 5, 12.8%), and other (*n* = 3, 7.7%). Drug prescription cases occurred in situations such as a doctor asking a clerk to enter a medication order into electronic health records on their behalf.

*When the staff member was asked by a doctor to type a drug order, the staff member mistyped the amount of Digoxin (digoxin is used to treat heart failure). (No. 6)*

Nine out of 24 cases consisted of mistakes in prescription information. For example, a reception clerk did not ask whether a patient was taking a particular medicine.

*Prior to the patient’s exam, the staff had asked the patient to stop taking the drug, but the patient didn't say anything, so the staff didn't specifically ask. On the day of the patient's exam, the patient was still on the medication. (No. 82)*

The next most common theme for direct impact on patient care was system administration, information, and documentation. Three out of seven cases were related to communication errors with another department.

*When I made a phone call regarding the preparation of chemotherapy, I said, “today's treatment will be done,” but it was wrong. I was supposed to say,“today's treatment was canceled.” (No 72)*

Mistakes in preparing exam documents and hospital ambulance management errors occurred in two cases. In Japan, several large hospitals have their own ambulances, which are managed and operated by hospital staff; accordingly, two cases related to ambulance equipment failure were reported.

**Table 1:** Direct impact on patient care

Code (theme)	Code (categories)	Code (meaning units; error/mistake)	***Examples***
Direct impact on patient care (*n* = 39, 45.9%)	Prescription drug (*n* = 24, 61.5%)	When a member of administrative staff writes a prescription by proxy on behalf of a doctor: ・error in medication name, dose, unit standard, or schedule (15 cases) ・error of wrong transcription of prescribed drugs (9 cases)	*The staff member was asked by a doctor to type a drug order and mistyped the amount of the drug. (No. 6)* *Prior to the patient’s exam, the staff member was supposed to ask the patient to stop taking the drug, but the patient did not say anything, so the staff member did not specifically ask. On the day of the patient’s exam, the patient was still on the medication. (No. 82)*
	System administration, information, and documentation. (*n* = 7, 17.9%)	When a member of the administrative staff uses an electronic (digital) hospital management system for information and documents: ・mistake in telephone call for treatment to another department (3 cases, including one misidentification case) ・mistake in transcribing exam documents (2 cases) ・error in management of hospital ambulance (2 cases)	*When I made a phone call regarding the preparation of chemotherapy, I said, “today’s treatment will be done,” but it was wrong. I was supposed to say, “today’s treatment was canceled.” (No. 72)* *The staff member forgot to document the items assessed during physical checkup. (No. 7)* *Ventilator did not connect to the oxygen valve in the ambulance. Repair technicians had confirmed that this valve connection was broken. (No. 66)*
	Inquiry (*n* = 5, 12.8%)	When a member of administrative staff works in hospital intake: ・error of measuring patient’s weight (3 cases) ・mistake in checking patient’s medical device record before exam (2 cases)	*The staff member measured patient’s height and weight, and transposed the height and weight on the electronic medical chart. The doctor prescribed the patient’s anti-cancer agent based on this mistyped record. (No. 68)*
	Other (*n* = 3, 7.7%)	・Error in acute patient care, administration of vaccine, and misidentification of patient’s meal (1 case each)	

Five out of the 39 (12.8%) direct care incidents were inquiry errors. Inquiry errors were usually directly related to treatment by a physician, such as before hemodialysis or a prescription-related weight measurement (3 cases). Additionally, in two cases, errors resulted from checking the patient’s medical device records or entering data into an electronic medical chart on behalf of a doctor. The following case illustrates how a serious accident may have occurred if a mistake with an anticancer drug had not been detected in advance.

*The staff member measured the patient's height and weight, but transposed height and weight in the electronic medical chart. The doctor prescribed the patient's anti-cancer agent based on this mistyped record. (No. 68)*

### Incident reports with indirect impact on patient care

Forty-six out of 85 (54.1%) cases represented an indirect impact on patient care, as shown in Table 2. These included five categories: system administration, information, and documentation (*n* = 22, 47.8%); reception (*n* = 9, 19.6%); reports of co-workers’ errors (*n* = 8, 17.4%); accounting (*n* = 6, 13.0%); and other (*n* = 1, 2.2%). The category of system administration, information, and documentation included misidentification of information and/or documents (e.g., fax number, patient profile, etc.) and database system errors (e.g., hospital electronic records, accounting management system).

*The staff put an incorrect blood type seal in the patient's chart. (No. 80)*

Hospital administrative staff frequently call and talk directly to patients through reception or accounting. In the reception category, examples of reported incidents include communication (four cases), misidentification of exam documents and administration of patient information (four cases), and miscommunication of forthcoming treatment (one case).

*The patient talked with administrative staff about having an MRI exam. The staff said it depended on the situation, but the patient came to the hospital because they thought they could have the MRI examination that day. (No. 6)*

The third most common type of incident report involving indirect care was a co-worker’s error (eight cases); doctor’s error was involved in five cases and nurses or nutritionists were report subjects in only one case. In accounting processes, there were six cases; errors in medical expenses occurred in four cases, and prescription accounting and misidentification of a patient’s ID card each occurred once.

**Table 2:** Indirect impact on patient care

***Code (theme)***	***Code (categories)***	***Code (meaning units; error/mistake)***	***Examples***
Indirect impact on patient care (*n* = 46, 54.1%)	System administration, information, and documentation (*n* = 22, 47.8%)	Error made by member of hospital administrative staff: ・misidentification of information and/or documents (e.g., fax number, patient profile, etc.) (7 cases) ・error in patients’ documents or database (7 cases) ・error in contact and coordination with other departments (4 cases) ・error in administration of electronic (digital) systems (4 cases)	*The staff put a blood type seal on the wrong patient’s chart. (No. 80)* *After the staff entered accounting management system data, the system was slow to process the data, so patients had to wait for a long time. (No. 34)*
	Reception (*n* = 9, 19.6%)	When a member of hospital administrative staff works in hospital reception: ・miscommunication (4 cases) ・misidentification of exam documents and administration of patient’s information (each 2 cases) ・entering incorrect treatment information by administrative staff member (1 case)	*The patient talked with the hospital staff about having an MRI. The staff said it depended on the situation, but the patient had come to the hospital because they thought they could have the MRI that day. (No. 6)*
	Reported another co-worker’s error (*n* = 8, 17.4%)	When a member of hospital administrative staff finds a co-worker’s error: ・doctor’s error (5 cases, including one misidentification case) ・nurse’s error (2 cases) ・nutritionist’s error (1 case)	*The staff received a call from a pharmacy about a doctor’s incorrect drug prescription. (No. 13)*
	Accounting (*n* = 6, 13.0%)	When a member of hospital administrative staff works in hospital accounting: ・accounting value error (4 cases) ・prescription accounting error (1 case) ・misidentification of patient’s ID card (1 case)	*The patient had to wait a long time because an error occurred in the accounting document. (No. 23)*
	Others (*n* = 1, 2.2%)	When a member of hospital administrative staff mails information to a nurse about medical checkups for hospital staff: ・misidentification due to nominal similarity of nurse’s name (1 case)	–

### Differences in case reports between direct and indirect care

Table 3 shows the frequency and percentage of case reports by various factors. The highest number of incidents was reported from January to March (*n* = 33, 38.8%), and the second highest was form October to November (*n* = 21, 24.7%). Almost all reported cases occurred in the daytime on weekdays (*n* = 79, 92.9%). Most direct-care incidents occurred from 2:00–3:00 pm (*n* = 11, 30.6%), while most indirect-care incidents occurred from 10:00–11:00 am (*n* = 14, 31.8%). The highest incident rates occurred in outpatient units (*n* = 23, 27.1%) and examination or operating rooms (*n* = 12, 14.1%).

**Table 3:** Frequency and percentage of case reports by various factors

	All	Direct care	Indirect care
	*n* = 85	*n* = 39	*n* = 46
Month			
Apr–Jun	18 (21.2)	9 (23.1)	9 (19.6)
July–Sep	13 (15.3)	7 (17.9)	6 (13.0)
Oct–Nov	21 (24.7)	9 (23.1)	12 (26.1)
Jan–Mar	33 (38.8)	14 (35.9)	19 (41.3)
Day			
Weekday	79 (92.9)	37 (94.9)	42 (91.3)
Weekend/Holiday	6 (7.1)	2 (5.1)	4 (8.7)
Time Period ^a^			
8 am–10 am	16 (20.0)	8 (22.2)	8 (18.2)
10 am–12 pm	20 (25.0)	6 (16.7)	14 (31.8)
12 pm–2 pm	11 (13.8)	5 (13.9)	6 (13.6)
2 pm–4 pm	19 (23.8)	11 (30.6)	8 (18.2)
4 pm–6 pm	11 (13.8)	5 (13.9)	6 (13.6)
6 pm–8 pm	2 (2.5)	1 (2.8)	1 (2.3)
8 pm–10 pm	1 (1.3)	0 (0.0)	1 (2.3)
Situation			
Outpatient	23 (27.1)	14 (35.9)	9 (19.6)
Examination/operation room	12 (14.1)	8 (20.5)	4 (8.7)
Wards	6 (7.1)	1 (2.6)	5 (10.9)
Pharmacy	3 (3.5)	2 (5.1)	1 (2.2)
Other	41 (48.2)	14 (35.9)	27 (58.7)

Number are shown as counts (frequencies)

^a^5 out of 85 answers were blank and were excluded from the analysis

Fourteen out of the 85 cases (16.5%) involved patient misidentification. Table 4 shows the incident report types and how these differed for direct-indirect care. The percentage of patient misidentification for indirect care (*n* = 12, 26.1%) was higher than that for direct care (*n* = 2, 5.1%). Furthermore, the number of incidents involving medication for direct patient care was more than twice that of indirect care.

**Table 4:** Incident report types

	All *n* = 85	Direct care *n* = 39	Indirect care *n* = 46
Patient misidentification			
Yes	14 (16.5)	2 (5.1)	12 (26.1)
No	71 (83.5)	37 (94.9)	34 (73.9)
Error types			
Medication	37 (43.5)	26 (66.7)	11 (23.9)
Surgery	1 (1.2)	0 (0.0)	1 (2.2)
Exam/treatment	8 (9.4)	5 (12.8)	3 (6.5)
Others	39 (45.9)	8 (20.5)	31 (67.4)

Numbers are shown as mean *n*(%)

## Discussion

In our results, incidents related to direct and indirect impact on patient care comprised similar percentages of cases, namely 46 and 54%, respectively. Task shifting might be affected by the changing roles of hospital administrative staff, as their work has changed from only administrative tasks to including some aspects of direct patient care. As no relevant data were collected until 10 years ago, we could not compare the current results with historical data. Nevertheless, our study suggests that administrative staff in hospitals have an important role in preventing errors within the medical team, as the work and roles of administrative staff are connected with patient safety.

The International Patient Safety Goals (IPSGs) were developed in 2006 by the International Joint Commission [16], and are monitored by accredited organizations. The three key goals pertaining to the errors identified in this study are discussed below.

### Goal 1: Identify patient correctly to prevent patient misidentification

Patient misidentification continues to be a serious problem in daily clinical practice, and can cause sentinel events. The Joint Commission defines sentinel events as patient safety events that result in death, permanent harm, or severe temporary harm [17]. Our findings revealed that patient misidentification, such as misidentification of information and documents including those related to a patient’s profile and treatment, was involved in 14 out of 85 cases. The WHO reported that the major areas where patient misidentification can occur include drug administration, phlebotomy, blood transfusions, and surgical interventions. Previous research has reported that limiting the working hours of clinical team members leads to an increased number of team members caring for each patient, thereby increasing the likelihood of hand-over and other communication problems [18]. In addition, the neonatal intensive care unit (NICU) has been identified as a major risk area for patient misidentification, due to the NICU patients frequently being similar in appearance and often not recognizably unique in terms of identifiers [19]. The present results suggest that misidentification errors occur in all hospital areas, including in reception and accounting. However, the most high-risk patient misidentifications by hospital administrative staff were in the category of indirect impact on patient care, such as system administration, information, and documentation.

It is common for inpatients to wear wristbands to prevent patient misidentification. However, outpatients do not wear wristbands in hospitals, thus causing patient misidentification during reception and accounting processes. Patient misidentification errors can be prevented when health care providers consistently verify patient identity using two unique patient identifiers, such as the wristband and verbal confirmation of the patient’s date of birth [20]. The need to verify patient identity applies to situations such as blood transfusion, surgery, and treatment. Abraham et al. [21] reported that among 293 reported incidents of patient misidentification, the most frequent errors were missing wristbands, wrong charts or notes in files, administrative issues, and wrong labeling in patient transfers.

In particular, administrative staff create and update the electronic charts that are used by all the members of medical teams. If the chart data are incorrect, the patient’s wristband, which displays important information such as the patient’s ID, would be incorrect. Our findings revealed that cases related to the patient’s ID included “error in patients’ documents or in database (system administration, information, and documentation in indirect care).” Clinical staff cross-reference the ID on the patient’s wristband with electronic medical record IDs in various clinical situations. Blood transfusion errors can cause catastrophic morbidity or mortality. Incorrect identification of patients is a major source of blood transfusion errors, and the methods to prevent this error include the use of wireless and bar-code technology [22]. Wristbands with patient information such as allergies [23] and falling risk [24] alert providers to high-risk patients. Severe incidents may occur if the administrator assigns the armband/wristband to the wrong patient. Hospital administrative staff need to recognize that their task is connected to identifying patients, and pay careful attention to performing this task correctly.

### Goal 2: Improve effective communication to prevent severe accidents and improve service

Administrative staff work everywhere in the hospital, such as the outpatient departments (reception and accounting), examination/operation room, ward, pharmacy, and the administrative floor of the hospital. Their work often involves contact and coordination with other departments. Our findings indicated that errors of communication contributed to three cases of direct impact on patient care in system administration, information, and documentation, and four cases of indirect impact on patient care involving miscommunication with patients in reception. Cases involving miscommunication led to long waiting periods for the patients or to hospitals suffering financial losses on drug cost. Hand-off is a transfer and acceptance of patient care responsibility achieved through effective communication. When hand-off communication fails, many factors are involved, such as health care provider training and expectations; language barriers; cultural or ethical considerations; and inadequate, incomplete or nonexistent documentation [25]. Hospital administrative staff need to supply communication training and education, because they often have the opportunity to connect and coordinate with patients, other occupational staff, another department, and medical staff outside of their institution.

### Goal 3: Improve the safety of high-alert medications to prevent medication error

Medication administration errors occur frequently and lead to patient harm, but there is weak evidence of the effectiveness of interventions in preventing such errors [26]. Evaluation of the types and frequencies of errors is of critical importance [27, 28]. It has been previously reported that 11% of all patients were subject to medication error, with risk factors including poor coordination of care, cost-related barriers to medical services or medicines, multi-morbidity, and hospitalization [21]. In our study, incidents related to drug prescription errors accounted for 62% of the incidents with a direct impact on patient care. The WHO reported that the factors associated with health care professionals that may influence medication errors are inadequate drug knowledge, inadequate prescription (e.g., an incorrect or incomplete prescription), lack of experience, and poor communication between health care professionals and with patients [28]. Many medications have similar names, which may lead to staff making medication-related errors. The reports of errors made by hospital administrative staff suggest role task-shifting by medical staff. However, although the role of medical assistants is to assist doctors, the education required to qualify as a medical assistant is insufficient in terms of learning all subject matter relevant to assisting physicians, particularly regarding drug-related knowledge. If medical assistants have insufficient knowledge, they cannot be aware of prescription mistakes. Previous medication error studies have focused on medical staff such as physicians, nurses, and pharmacists. However, our findings suggest that the role of administrative staff is diverse and includes elements that determine critical treatment decisions. For example, measuring weight, which falls within the scope of the role of administrative staff, is used to decide dosage for medications such as anti-cancer drugs, or the quantity of fluid to remove during dialysis. Mistakes made in measuring or transcription might lead to weight-based over- or under-dosing decisions. Hospital administrative staff should be aware of working with high-alert medicines that require a high degree of caution, and properly handing of information and/or documents to physicians. Due to limited secondary data, a root cause analysis could not be performed to determine why physicians at times omitted the reconfirmation process. If physicians consider administrative staff to be medical staff, hospital managers must educate physicians so that they can distinguish between administrative staff and health care professionals.

### Limitations

The present study has some limitations. First, although we used data gathered over a period of 10 years, the study sample was small. From January to December 2017, 5129 cases were reported to JCQHC, with 45% reported by physicians and 49% reported by nurses [13]; only 85 incidents were reported by hospital administrative staff. Second, we used secondary data collected from JCQHC incident reports, which did not include the reporters’ characteristics and institutional information. Clinical staff are generally guaranteed full-time and permanent employment, while administrative staff have various employment contracts; most are temporary, part-time employees. JCQHC data do not provide employment status information, so we could not segregate the incident reports according to those reported by full-time and part-time workers.

## Conclusions

Physician shortage is a common problem in developed countries. In response to this issue, the Japanese government’s system reforms include shifting tasks from physicians to hospital administrative staff. Prior studies of medical errors have focused on medical staff, such as physicians, nurses, and pharmacists, as they provide direct patient care. The current study found that major errors were reported by hospital administrative staff, including errors in entering prescription drug information on behalf of a physician, misidentification when indirectly involved with patient care, and errors in system administration, information, and documentation. Although this study has some limitations, such as a small sample size and the use of secondary data, it is one of the few studies to examine incidents reported by hospital administrative staff.

## Data Availability

We used JCQHC open data. The datasets used and/or analyzed during the current study are available from the corresponding author upon reasonable request.

## Abbreviations

AAMC: Association of American Medical Colleges
JCQHC: Japan Council for Quality Healthcare
